# PRISMA-compliant meta-analysis: association of metabolic syndrome and its components with the risk of chronic obstructive pulmonary disease

**DOI:** 10.1042/BSR20181199

**Published:** 2018-11-28

**Authors:** Linyang Ye, Xi Huang, Qingxiang Wang, Hualing Yang, Dongmiao Cai, Zhanxiang Wang

**Affiliations:** 1 Department of Anesthesiology, The First Affiliated Hospital of Xiamen University, Xiamen, Fujian Province, P.R. China; 2 The First Clinical Medical College, Fujian Medical University, Fuzhou, Fujian Province, P.R. China; 3 Department of Neurosurgery, The First Affiliated Hospital of Xiamen University, Xiamen, Fujian Province, P.R. China

**Keywords:** association, chronic obstructive pulmonary disease, metabolic syndrome, meta-analysis

## Abstract

A preferred reporting items for systematic reviews and meta-analyses-compliant meta-analysis was conducted to test the association of metabolic syndrome and its components with the risk of chronic obstructive pulmonary disease (COPD) based on observational studies. Literature retrieval, article selection and data extraction were done by two researchers independently. Total 16 articles (20 independent studies) were analyzed with 3915 COPD patients and 25,790 control participants. Overall analysis indicated that metabolic syndrome was significantly associated with 1.53-fold (95% confidence interval [CI]: 1.23–1.9, *P*<0.001) increased risk of COPD, with moderate heterogeneity (*I*^2^ = 74.3%). Of four metabolic components, hypertension was significantly associated with 1.55-fold (95% CI: 1.14–2.11, *P*=0.005) increased risk, and averaged levels of systolic blood pressure (weighted mean difference [WMD] = 3.626 mmHg, 95% CI: 1.537–5.714, *P*<0.001) and glucose (WMD = 2.976 mmol/l, 95% CI: 0.141–5.812; *P*=0.04) were significantly higher in COPD patients than in control participants, yet that of body mass index (WMD = −1.463 kg/m^2^, 95% CI: −2.716 to −0.211, *P*=0.022) were significantly lower. Gender, race, source of control participants, matched status and sample size were identified as accountable factors for significant heterogeneity. Altogether, the presence of metabolic syndrome, especially its component hypertension, was associated with significantly increased risk of COPD.

## Introduction

Chronic obstructive pulmonary disease (COPD) is a systemic inflammatory disorder characterized by airflow obstruction. COPD is the leading cause of mortality worldwide, as it afflicts approximately 174.5 million adults [1] and leads to 3.2 million deaths [2] in 2015. The incidence rate of COPD is on the rise, mainly due to ambient air pollution, high cigarette smoking, childhood chronic cough, low awareness and the lack of early detection [3–6]. Hence, pre-identifying individuals at high-risk for COPD may result in improved prevention and early detection. A growing emphasis should be put on clinical epidemiology research and the translation of research findings into advances in screening and clinical care.

It is widely recognized that systemic inflammation plays a central role in the pathogenesis of COPD, yet the exact mechanism remains incompletely understood [7,8]. As a low-grade systemic inflammatory condition, metabolic syndrome has been acknowledged as a common comorbidity of COPD [9]. Some investigators claimed that metabolic syndrome was more frequent in COPD patients than healthy cohorts [10,11]; however, others failed to support this claim [12,13]. A recent systematic review was written by Cebron Lipovec and colleagues who summarized the prevalence of metabolic syndrome in COPD and found that 32% of COPD patients had metabolic syndrome, 2.0 per cent points significantly higher than control participants [14], yet they had not interrogated the contribution of metabolic syndrome to the risk of having COPD. However, literature retrieval failed to reveal any evidence on this risk prediction. To produce more information, we sought to undertake a meta-analysis to test the association of metabolic syndrome with COPD risk by pooling the results of 16 articles.

This meta-analysis of observational studies was conducted according to the guidelines reported in the Preferred Reporting Items for Systematic Reviews and Meta-analyses (PRISMA) statement [15] (PRISMA checklist in Supplementary Table S1).

## Methods

### Literature retrieval

Three public databases, PubMed, EMBASE and Google/Scholar, were reviewed prior to January 14, 2018 to retrieve eligible articles on the association between metabolic syndrome and COPD from the English-language literature. Terms used for literature retrieval were all MeSH terms, including (‘pulmonary disease, chronic obstructive’ OR ‘chronic obstructive pulmonary disease’) AND (‘metabolic syndrome’) AND (‘prevalence’ OR ‘comorbidity’). Literature search was additionally extended to the reference lists of retrieved articles to avoid possible missing hits.

### Inclusion and exclusion criteria

Assessment of each article was based on both inclusion and exclusion criteria. Inclusion criteria that must be met simultaneously incorporated (i) original data analysis, (ii) either cross-sectional or nested case–control study design, (iii) clear diagnoses of metabolic syndrome and COPD, (iv) accessible number of cases with metabolic syndrome between COPD patients and control participants. Meanwhile, exclusion criteria incorporated (i) publication in form of abstracts due to inadequate data for a complete comparison, (ii) case reports or case series, (iii) systematic reviews or meta-analyses, (iv) lack of control participants.

### Article selection

Selection process was completed independently by the two researchers (Hualing Yang and Xi Huang), who justified the eligibility of each article through reading title or abstract, and full-text if necessary. Any uncertainty or ambiguity, if exists, was discussed by the two researchers or adjudicated by a third researcher (Zhanxiang Wang), and if necessary original researchers were contacted for confirmation by e-mails. A consensus was reached at the end.

### Data extraction

From each eligible article, the following data were extracted: the first author’s name, year of publication, study design, source of control participants, country where participants resided, onset age of COPD diagnosed, diagnostic criteria of COPD and metabolic syndrome, ascertainment of control participants, matched condition between patients and controls, sample size, age, gender, body mass index (BMI), waist circumstance, the percentage of metabolic syndrome in patients and controls, cigarette smoking, pack-year smoking history, alcohol drinking, physical activity, income level, education level, forced expiratory volume in one second (FEV1), FEV1 to forced vital capacity (FVC) ratio, systolic blood pressure (SBP), diastolic blood pressure (DBP), glucose, triglycerides, total cholesterol (TC), high-density lipoprotein cholesterol (HDLC), low-density lipoprotein cholesterol (LDLC), hypertension, diabetes mellitus, dyslipidemia, lipid-lowing therapy, anti-hypertensive treatment and anti-diabetic treatment. If data were provided separately by gender, each was presented and analyzed individually. Data abstraction was independently completed by the two researchers (Hualing Yang and Xi Huang) according to a uniform design table, and data were computerizedly checked for uniformity between the two tables. Any divergence was solved by reviewing original context until a consensus was reached.

### Quality assessment

The quality of each eligible study was assessed by the Newcastle–Ottawa Scale (NOS) for case–control studies, and the NOS is an ongoing collaboration between the Universities of Newcastle, Australia and Ottawa, Canada.

### Statistical analysis

Risk effect estimates for metabolic syndrome and its categorical components in association with COPD were expressed as odds ratio (OR) and 95% confidence interval (95% CI) under a random-effects model by using the DerSimonian and Laird method [16]. Mean differences in BMI, waist circumstance, FEV1, FEV1 to FVC ratio, SBP, DBP, glucose, triglycerides, TC, HDLC and LDLC between COPD patients and controls were expressed as weighted mean difference (WMD) and 95% CI under a random-effects model.

The *I*^2^ statistic was calculated to quantify the magnitude of between-study heterogeneity, and statistical significance was reported if this statistic exceeds 50%. Subgroup analyses by diagnostic criteria of metabolic syndrome, gender, source of control participants, race, country development, matched status and sample size were carried out to see whether these grouping factors can account for between-study heterogeneity. Meta-regression analyses were also conducted to explore other sources of between-study heterogeneity.

Cumulative analysis was conducted to identify the effect of the first published study on the following publications, and to inspect the evolution of cumulated estimates over time. Influential analysis was also conducted to check the effect of any individual studies on the overall estimates.

Begg’s and filled funnel plots (visual appraisal of symmetry) and Egger’s tests at a significance level of 10% were used to assess the likelihood of publication bias. The number of theoretically missing studies with negative results or in small scales can be depicted in the filled funnel plot through the trim-and-fill method.

The STATA/SE version 14.1 (StataCorp LP, College Station, Texas) was used to manage data and figures. Study power was estimated using the PS Power and Sample Size Calculations version 3.0 [17].

## Results

### Eligible studies

In total, 674 articles were identified from three public databases using predefined key words, only 16 articles met our inclusion and exclusion criteria [10–13,18–29], and the selection process is illustrated in Supplementary Figure S1. Four articles that presented gender-specific data were treated separately [11,22,27,29], and so there were 20 independent studies in the final analysis, including 3915 COPD patients and 25,790 control participants. As for quality assessment, total NOS score ranged from 5 to 8, with a mean value of 6.31. The baseline characteristics of all eligible studies are shown in Table 1.

**Table 1:** The baseline characteristics of all eligible studies in this meta-analysis

Author	Year	Study design	Control source	Country	Matched	COPD diagnosis	MetS diagnosis	Sample size		Mean age (years)		Male gender		Mean BMI (kg/m^2^)		Mean FEV1 (%)		Mean FEV1 to FVC ratio		MetS		NOS score
								Cases	Controls	Cases	Controls	Cases	Controls	Cases	Controls	Cases	Controls	Cases	Controls	Cases	Controls	
Gupta (ATP III) [10]	2017	Cross-sectional	Hospital	India	Yes	GOLD	ATP III	90	45	0.689	0.578	23.29	22.59	NA	NA	NA	NA	0.156	0.000	6	53.1	54.5
Gupta (IDF) [10]	2017	Cross-sectional	Hospital	India	Yes	GOLD	IDF	90	45	0.689	0.578	23.29	22.59	NA	NA	NA	NA	0.333	0.000	6	53.1	54.5
Waschki [18]	2016	Nested	Hospital	Germany	Yes	GOLD	IDF	74	18	0.703	0.611	26.00	25.70	55.20	116.20	51.00	78.60	0.473	0.333	6	66.0	65.9
Munoz-Esquerre [19]	2016	Nested	Hospital	Spain	NA	GOLD	JIS (2009)	17	14	0.941	0.929	24.00	27.10	62.00	97.70	54.90	76.30	0.353	0.786	6	63.4	58.3
Bozek [20]	2016	Cross-sectional	Population	Poland	Yes	GOLD	ICD-10	1084	1076	0.680	0.410	21.40	31.30	66.30	90.30	NA	NA	0.251	0.136	8	66.5	68.6
Acharyya (ATP III) [21]	2016	Cross-sectional	Population	India	Yes	GOLD	ATP III	77	77	0.740	0.740	23.00	24.00	NA	NA	NA	NA	0.442	0.312	7	60.0	60.0
Acharyya (IDF) [21]	2016	Cross-sectional	Population	India	Yes	GOLD	IDF	77	77	0.740	0.740	23.00	24.00	NA	NA	NA	NA	0.312	0.325	7	60.0	60.0
Chung (Male) [22]	2015	Nested	Population	Korea	NA	GOLD	ATP III	760	2346	1.000	1.000	23.50	24.30	77.10	96.30	NA	NA	0.295	0.264	6	64.5	53.2
Chung (Female) [22]	2015	Nested	Population	Korea	NA	GOLD	ATP III	279	3731	0.000	0.000	23.30	24.10	75.70	98.50	NA	NA	0.380	0.320	6	64.5	55.4
Park [23]	2014	Cross-sectional	Population	U.S.A.	NA	GOLD	JIS (2009)	94	3661	0.447	0.511	26.98	29.30	67.00	96.00	58.00	76.00	0.575	0.536	6	62.1	56.6
Breyer [24]	2014	Cross-sectional	Population	Netherlands	NA	GOLD	IDF	228	156	0.590	0.450	26.20	27.30	52.80	120.40	40.90	78.10	0.570	0.400	6	63.7	60.1
Ozgen [25]	2013	Cross-sectional	Population	Turkey	Yes	GOLD	IDF	50	40	0.900	0.850	27.20	27.60	46.30	NA	53.00	NA	0.440	0.300	8	61.3	58.4
Hosny [26]	2013	Cross-sectional	Hospital	Egypt	Yes	GOLD	ATP III	50	35	0.880	0.914	27.00	28.00	54.30	NA	62.20	NA	0.400	0.171	7	57.7	55.9
Park (Male) [27]	2012	Nested	Population	Korea	NA	GOLD	ATP III	100	437	1.000	1.000	23.30	24.10	NA	NA	NA	NA	0.330	0.222	6	60.9	50.8
Park (Female) [27]	2012	Nested	Population	Korea	NA	GOLD	ATP III	33	645	0.000	0.000	24.20	24.10	NA	NA	NA	NA	0.485	0.296	6	59.2	51.4
Akpinar [28]	2012	Nested	Hospital	Turkey	Yes	GOLD	ATP III	91	42	0.857	0.833	NA	NA	NA	NA	NA	NA	0.446	0.171	7	63.7	62.8
Lam (Male) [29]	2010	Nested	Population	China	NA	GOLD	IDF	128	1880	1.000	1.000	NA	NA	NA	NA	NA	NA	0.226	0.198	6	67.1	63.5
Lam (Female) [29]	2010	Nested	Population	China	NA	GOLD	IDF	368	4982	0.000	0.000	NA	NA	NA	NA	NA	NA	0.226	0.198	6	62.7	60.7
Funakoshi [12]	2010	Cross-sectional	Population	Japan	NA	GOLD	ATP III	297	6544	1.000	1.000	22.70	23.70	89.00	95.80	66.10	79.50	0.168	0.258	6	62.3	55.9
Watz [13]	2009	Cross-sectional	Hospital	Germany	NA	GOLD	IDF	57	30	0.719	0.767	27.80	27.50	63.00	99.60	53.20	75.00	0.526	0.533	5	63.3	62.6
Marquis (Male) [11]	2005	Cross-sectional	Hospital	Canada	Yes	ATS (1987)	ATP III	23	20	1.000	1.000	29.00	30.00	NA	NA	NA	NA	0.609	0.200	8	66.0	63.0
Marquis (Female) [11]	2005	Cross-sectional	Hospital	Canada	Yes	ATS (1987)	ATP III	15	14	0.000	0.000	27.00	29.00	NA	NA	NA	NA	0.267	0.214	8	66.0	63.0

Abbreviations: ATP-III, the Adult Treatment Panel III; ATS (1987), American Thoracic Society in 1987; BMI, body mass index; COPD, chronic obstructive pulmonary disease; FEV1, forced expiratory volume in one second; FVC, forced vital capacity; IDF, International Diabetes Federation; JIS (2009), a joint interim statement in 2009 (Circulation 2009; 120:1640–1645); MetS, metabolic syndrome; NA, not available; NOS, Newcastle–Ottawa Scale.

### Overall analyses – metabolic syndrome and COPD

**Figure 1:**
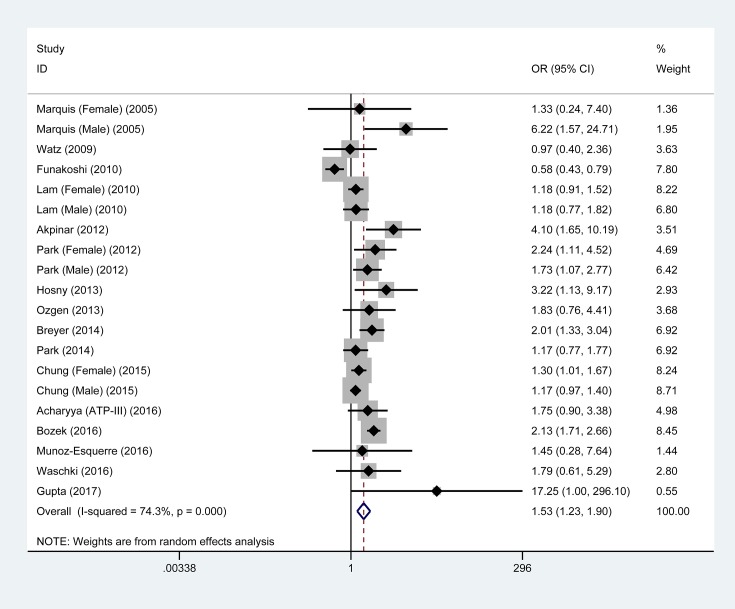
Overall association of metabolic syndrome with COPD risk Abbreviations: 95% CI, 95% confidence interval; COPD, chronic obstructive pulmonary disease; OR, odds ratio. OR is denoted by the center of a solid diamond, and the length of solid line cross this diamond denotes its 95% CI. The hollow diamond with a vertical broken line denotes overall risk estimate. The solid vertical line is set at the null value (OR = 1.0).

As shown in Figure 1, metabolic syndrome was significantly associated with 1.53-fold (95% CI: 1.23–1.90, *P*<0.001) increased risk of COPD when pooling the results of 20 eligible studies together. The *I*^2^ statistic was 74.3%, indicating moderate between-study heterogeneity. With an estimated prevalence of COPD of 13.5% for adults aged 20–79 years old [30], the power to derive an OR of 1.53 in 3915 COPD patients and 25,790 control participants was 100% using a 2-sided alpha of 5%.

The results of cumulative and influential analyses are presented in Supplementary Figure S2. In the cumulative analysis, the overall estimation tended to be stabilized since 2012, and significance was reached after 2013. In the influential analysis, no single study was noted to affect the overall estimation remarkably.

### Overall analyses – metabolic components and COPD

Table 2 presents the association of metabolic components with COPD using available data from all eligible studies. At a categorical scale, hypertension was significantly associated with 1.55-fold increased risk of COPD (95% CI: 1.14–2.11, *P*=0.005), while the risk conferred by obesity was marginally reduced by 32% (95% CI: 0.46–1.01, *P*=0.057). No hints of significance were observed for diabetes mellitus, high waist circumstance, high triglycerides and low HDLC (*P*>0.05). When metabolic components were analyzed continuously, averaged levels of SBP (WMD = 3.626 mmHg, 95% CI: 1.537–5.714, *P*<0.001) and glucose (WMD = 2.976 mmol/l, *P*=0.040) were significantly higher in COPD patients than in controls, yet that of BMI (WMD = −1.463 kg/m^2^, 95% CI: −2.716 to −0.211, *P*=0.022) were significantly lower. There was no significance for the other continuous variables (*P*>0.05).

**Table 2:** Effect estimates of metabolic components in association with COPD

Metabolic components	Number	EE	95% CI	*P*	*I*^2^	*P*_Q-test_	Egger’s *P*
Categorical scale (EE = OR)							
Hypertension	11	1.55	1.14–2.11	0.005	64.4%	0.002	0.539
Diabetes	12	1.1	0.93–1.32	0.273	13.5%	0.312	0.01
Obesity	7	0.68	0.46–1.01	0.057	81.6%	<0.001	0.243
High WC	9	1.19	0.78–1.79	0.42	73.0%	<0.001	0.006
High triglycerides	10	1.28	0.9–1.81	0.169	66.2%	0.002	0.063
Low HDLC	10	1.09	0.82–1.45	0.536	26.8%	0.197	0.568
Continuous scale (EE = WMD)							
BMI	17	–1.463	–2.716 to –0.211	0.022	98.2%	<0.001	0.728
WC	16	0.247	–0.666 to 1.16	0.596	72.3%	<0.001	0.101
SBP	15	3.626	1.537 to 5.714	0.001	81.7%	<0.001	0.786
DBP	15	–0.708	–1.842 to 0.426	0.221	80.9%	<0.001	0.574
Glucose	15	2.976	0.141 to 5.812	0.04	81.8%	<0.001	0.025
Triglycerides	17	–4.827	–10.685 to 1.031	0.106	68.0%	<0.001	0.706
TC	5	–2.385	–10.346 to 5.701	0.563	86.4%	<0.001	0.809
HDLC	17	0.234	–0.825 to 1.293	0.665	63.2%	<0.001	0.248
LDLC	7	–3.609	–12.038 to 4.82	0.401	87.6%	<0.001	0.912

Abbreviations: 95% CI, 95% confidence interval; BMI, body mass index; COPD, chronic obstructive pulmonary disease; DBP, diastolic blood pressure; EE, effect estimate; HDLC, high-density lipoprotein cholesterol; LDLC, low-density lipoprotein cholesterol; OR, odds ratio; SBP, systolic blood pressure; TC, total cholesterol; TG, triglycerides; WC, waist circumstance; WMD, weighted mean difference. The term ‘Number’ in the first row referred to the number of eligible studies.

### Pulmonary functional testing

**Figure 2:**
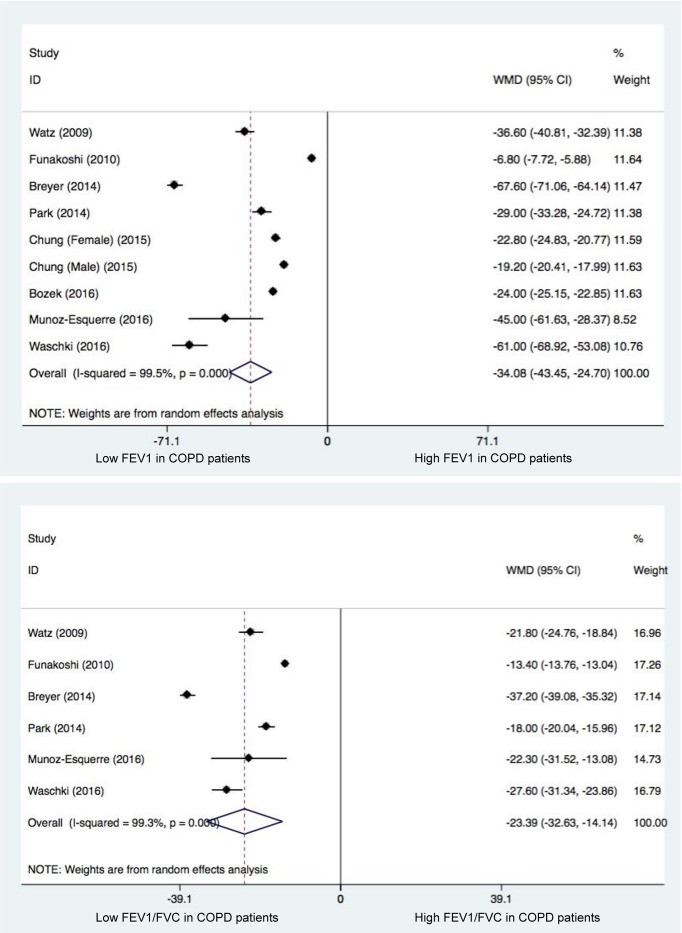
Changes of FEV1 (the upper panel) and FEV1 to FEV ratio (the lower panel) between COPD patients and control participants Abbreviations: 95% CI, 95% confidence interval; COPD, chronic obstructive pulmonary disease; FEV1, forced expiratory volume in one second; FVC, forced vital capacity; WMD, weighted mean difference. WMD is denoted by the center of a solid diamond, and the length of solid line cross this diamond denotes its 95% CI. The hollow diamond with a vertical broken line denotes overall risk estimate. The solid vertical line is set at the null value (WMD = 0.0).

Two indices of pulmonary functional testing, FEV1 and FEV1 to FVC ratio, were summarized and compared between COPD patients and controls (Figure 2). As expected, the two indices were remarkably lower in COPD patients than in control participants (*P*<0.001).

### Stratified analyses – metabolic syndrome and COPD

Stratified effect estimates of metabolic syndrome for COPD risk are presented in Table 3. By COPD diagnosis, metabolic syndrome was significantly associated with COPD in studies adopting the GOLD criteria (OR = 1.48, 95% CI: 1.20–1.84, *P*<0.001), but not in studies adopting the American Thoracic Society (ATS) criteria in 1987. By the diagnostic criteria of metabolic syndrome, most studies adopted the criteria developed by ATP-III (Adult Treatment Panel III) and IDF (International Diabetes Federation), and the association of metabolic syndrome with COPD was statistically significant using the two major criteria (OR = 1.72 and 1.39, 95% CI: 1.24–2.4 and 1.03–1.88, *P*=0.001 and 0.032, respectively). By gender, the risk prediction of metabolic syndrome for COPD was only significant in females (OR = 1.28, 95% CI: 1.08–1.53, *P*=0.004). By study design, significance was attained in studies of both cross-sectional and nested designs (OR = 1.67 and 1.35, 95% CI: 1.09–2.58 and 1.14–1.6, *P*=0.02 and <0.001, respectively). By source of controls, effect estimate was significant for both hospital-based and population-based sources, especially in hospital-based controls (OR = 2.43, 95% CI: 1.41–4.18, *P*=0.001). By race, the risk of COPD was significant in Caucasian and Middle-Eastern populations (OR = 2.05 and 2.83, 95% CI: 1.7–2.46 and 1.65–4.86, both *P*<0.001). By development, the association of metabolic syndrome with COPD was significant in both developed and developing countries (OR = 1.44 and 1.76, 95% CI: 1.09–1.9 and 1.19–2.6, *P*=0.01 and 0.004, respectively). By matched status, studies with matched patients and controls reported a significant association of metabolic syndrome with COPD (OR = 2.21, 95% CI: 1.83–2.68, *P*<0.001). Finally, by sample size, the risk estimate was 2.16 (95% CI: 1.47–3.16) in small studies (total sample size <300) and 1.34 (95% CI: 1.05–1.71) in large studies (total sample size ≥300) (*P*<0.001 and *P*=0.02, respectively).

**Table 3:** Stratified effect estimates of metabolic syndrome for COPD risk

Stratified groups	Number	OR	95% CI	*P*	*I*^2^	*P*_Q-test_
COPD diagnosis						
GOLD	18	1.48	1.20–1.84	<0.001	75.4%	<0.001
ATS (1987)	2	3.14	0.70–14.09	0.134	46.9%	0.170
By MetS						
ATP-III	12	1.72	1.24–2.4	0.001	83.6%	<0.001
IDF	8	1.39	1.03–1.88	0.032	49.1%	0.056
JIS (2009)	2	1.18	0.79–1.77	0.41	0.0%	0.802
By gender						
Males	5	1.22	0.78–1.91	0.384	84.8%	<0.001
Females	4	1.28	1.08–1.53	0.004	0.0%	0.414
By study design						
Cross-sectional	11	1.67	1.09–2.58	0.02	83.2%	<0.001
Nested	9	1.35	1.14–1.6	<0.001	34.5%	0.142
By source of controls						
Hospital	8	2.43	1.41–4.18	0.001	33.7%	0.159
Population	12	1.38	1.1–1.73	0.006	80.6%	<0.001
By race						
Asian	9	1.24	0.97–1.59	0.085	74.2%	<0.001
Caucasian	7	2.05	1.7–2.46	<0.001	0.0%	0.445
Middle Eastern	3	2.83	1.65–4.86	<0.001	0.0%	0.440
Mixed	1	1.17	0.77–1.77	0.46	NA	
By country development						
Developed	13	1.44	1.09–1.9	0.01	80.0%	<0.001
Developing	7	1.76	1.19–2.6	0.004	57.5%	0.028
By matched status						
NR	11	1.23	1.0–1.52	0.051	69.7%	<0.001
Yes	9	2.21	1.83–2.68	<0.001	0.0%	0.460
By total sample size						
<300	10	2.16	1.47–3.16	<0.001	19.6%	0.262
≥300	10	1.34	1.05–1.71	0.02	83.8%	<0.001

Abbreviations: 95% CI, 95% confidence interval; ATP-III, the Adult Treatment Panel III; COPD, chronic obstructive pulmonary disease; IDF, International Diabetes Federation; JIS (2009), a joint interim statement in 2009 (Circulation 2009; 120:1640–1645); MetS, metabolic syndrome; NA, not reported; OR, odds ratio. The term ‘Number’ in the first row referred to the number of eligible studies.

In stratified analyses, heterogeneity was non-significant in studies with the diagnosis of metabolic syndrome using IDF and JIS-2009 (*I*^2^ = 49.1% and 0.0%, respectively), involving female gender only (*I*^2^ = 0.0%), with nested design (*I*^2^ = 34.5%), involving hospital-based controls (*I*^2^ = 33.7%), including Caucasian or Middle Eastern populations (both *I*^2^ = 0.0%), with matched patients and controls (*I*^2^ = 0.0%) and with total sample size less than 300 (*I*^2^ = 19.6%).

### Meta-regression analyses

Besides stratified analyses, meta-regression analyses were conducted to explore other potential sources of heterogeneity for the association of metabolic syndrome with COPD by modelling age, gender, smoking, pack-year smoking history, drinking, physical activity, income and education. None of these factors exhibited significant contributions to the association between metabolic syndrome and COPD (all *P*>0.1).

### Publication bias

**Figure 3:**
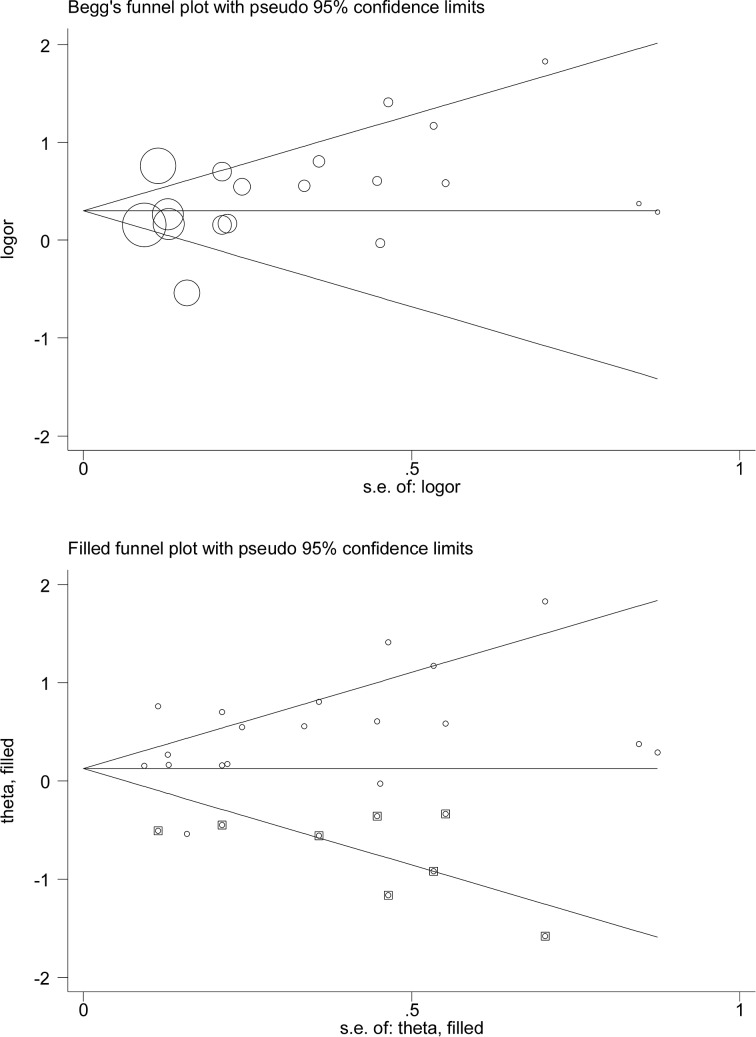
Begg’s (the upper panel) and filled (the lower panel) funnel plots for the association of metabolic syndrome with COPD risk Abbreviations: COPD, chronic obstructive pulmonary disease; logor, the logarithm of odds ratio; S.E., standard error. In Begg’s funnel plot, the symbols denoting the data in the plot are sized proportionally to inverse variance. In filled funnel plot, hollow circles denote the actual studies included in this meta-analysis, and solid squares denote missing studies required to achieve symmetry of funnel plot.

For metabolic syndrome in association with COPD, there was a low probability of publication bias, as reflected by Begg’s and filled funnel plots (Figure 3), although there were an estimated eight missing studies to make the filled funnel plot symmetrical. After considering the impact of missing studies, the association between metabolic syndrome and the risk of COPD was still statistically significant (OR = 1.13, 95% CI: 1.05–1.23, *P*=0.002).

For the association of metabolic components with COPD, the probability of publication bias was significant for diabetes mellitus, high waist circumstance, high triglycerides and glucose at a significance level of 10% (Egger’s *P*=0.01, 0.006, 0.063 and 0.025, respectively). There was no evidence of publication bias for other metabolic components, either categorical or continuous (Egger’s *P*>10%).

## Discussion

The aim of this meta-analysis is to test the association of metabolic syndrome and its components with COPD risk. Importantly, our data indicate that the comorbidity of metabolic syndrome, especially its component hypertension, was associated with significant risk of experiencing COPD. Moreover, gender, race, source of control participants, matched status and sample size were accountable factors for significant heterogeneity. To our knowledge, this is the first quantitative assessment of metabolic syndrome and COPD in the medical literature.

Metabolic syndrome as a potential risk factor for the development of COPD has been widely evaluated, while no consensus has been attained yet, mainly because of racial diversity of study populations, insufficient power of some individual studies and heterogeneous methodological designs [13,23]. A recent systematic review concluded that the prevalence of metabolic syndrome was significantly higher in COPD patients than controls, and hypertension, abdominal obesity and hyperglycemia constituted the most prevalent components [14]. However, to what magnitude metabolic syndrome contributes to the occurrence of COPD has not yet been clarified in this review [14]. Moreover, it is worth noting that metabolic syndrome is significantly more common in non-emphysematous COPD [31], which is defined by airflow obstruction with a paucity of emphysema on chest CT scan. However, considering that only one [19] of all eligible studies in this meta-analysis provided data on emphysema in COPD patients and control participants, we here focused on COPD irrespective of emphysema phenotype. To yield more information, we, in a larger sample size, found that patients with metabolic syndrome were about 1.5 times more like to develop COPD, and especially its component hypertension played a dominant role in predicting the disease risk. Additionally, we observed that COPD patients tended to have a lower level of BMI, which differed from that reported in this previous review [14]. In support of our findings, a large number of studies have demonstrated that low BMI was not only a high-risk factor for COPD [24,32–35], but also a significant predictor for all-cause mortality in COPD patients [36–38]. There is evidence that low BMI was associated with systemic inflammation that served as one of the possible mechanisms in COPD patients [39]. It is widely recognized that COPD is characterized by low-grade systemic inflammation that affects body composition [40], and systemic inflammation can promote insulin resistance and further contribute to the development of metabolic syndrome [41,42]. In addition, some systemic inflammatory markers such as leukocyte count [43] were observed to be significantly higher in COPD patients with metabolic syndrome than in patients without metabolic syndrome. It is suggested that several factors may contribute to the co-existing COPD and metabolic syndrome, including the presence of physical inactivity and systemic inflammation related to a smoking habit, sedentary lifestyle, airway inflammation and obstruction, adipose tissue and inflammatory marker activation [44]. Based on above lines of evidence, it is reasonable to postulate that the COPD risk conferred by metabolic syndrome might be mediated through an inflammation-related process. An in-depth assessment of this process is beyond the scope of this present study but certainly warrants further investigations.

Another key finding of this meta-analysis is that in stratified analysis, gender, race, source of control participants, matched status and sample size were identified as accountable factors for significant between-study heterogeneity. In particular, we found that the magnitude of COPD risk conferred by the presence of metabolic syndrome was markedly reinforced when analysis was restricted to females, Caucasian populations, studies involving hospital-based controls and studies of matched patients and controls, respectively. The reasons for differed risk magnitude are multifaceted, relating either to different exposures to environmental and lifestyle factors (such as cigarette smoking) or to different genetic backgrounds across races or ethnicities. For example, cigarette smoking is a predisposing factor for the development of both metabolic syndrome and COPD, and predisposition to smoking varies by gender [45,46]. Moreover, although sample size can explain some heterogeneity, the association between metabolic syndrome and COPD remained significant when analysis was restricted to larger studies in this study, suggesting the robustness of our findings. Nevertheless, to derive a more reliable estimate, we agree that some large, soundly designed, prospective studies are required.

## Limitations

The first limitation is that the association between metabolic syndrome and COPD does not prove causality. The second limitation revolves around the use of published aggregate data, not individual participant data, limiting further explorations, such as the three-way interaction between smoking, metabolic syndrome and COPD, which could provide clinically relevant insights into the increased risk of COPD among susceptible individuals. The third limitation is that the exclusion of non-English research papers, might lead to exaggerated estimates of observed association. The fourth limitation is that we did not consider the impact of therapies (especially steroids) on the association between metabolic syndrome and COPD due to sparse data. The fifth limitation is that this meta-analysis involved studies in varying definitions regarding both metabolic syndrome and COPD, which might produce a misclassification bias.

## Conclusions

Taken together, in a quantitative analysis of 29,705 subjects, our data indicate that the presence of metabolic syndrome, especially its component hypertension, was associated with significantly increased risk of COPD. Moreover, gender, race, source of control participants, matched status and sample size were accountable factors for significant heterogeneity. So for practical reasons, prevention and early detection of COPD patients prone to the development of metabolic syndrome and its sequelae may facilitate intensive surveillance or targeted interventions for high-risk patients, and thereby be of significant clinical importance.

## Supporting information

**Supporting Figure 1:** PRISMA flow chart for article selection in this meta-analysis

**Supplementary Figure 2:** Cumulative (the left panel) and influential (the right panel) analyses for overall association of metabolic syndrome with the risk of chronic obstructive pulmonary disease. In the lower panel, 1 denotes the study by Marquis et al in females, 2 denotes denotes the study by Marquis et al in males, 3 denotes the study by Watz et al, 4 denotes the study by Funakoshi et al, 5 denotes the study by Lam et al in females, 6 denotes the study by Lam et al in males, 7 denotes the study by Akpinar et al, 8 denotes the study by Park et al in females in 2012, 9 denotes the study by Park et al in males in 2012, 10 denotes the study by Hosny et al, 11 denotes the study by Ozgen et al, 11 denotes the study by Breyer et al, 12 denotes the study by Park et al in 2014, 13 denotes the study by Chung et al in females, 14 denotes the study by Chung et al in males, 15 denotes the study by Acharyya et al, 16 denotes the study by Bozek et al, 17 denotes the study by Munoz-Esquerre et al, 18 denotes the study by Waschki et al and 19 denotes the study by Gupta et al. Abbreviations: 95% CI, 95% confidence interval.

**Supplementary Table 1:** The PRISMA checklist

## Abbreviations

BMI: body mass index
COPD: chronic obstructive pulmonary disease
DBP: diastolic blood pressure
FEV1: forced expiratory volume in one second
FVC: FEV1 to forced vital capacity
HDLC: high-density lipoprotein cholesterol
LDLC: low-density lipoprotein cholesterol
NOS: Newcastle–Ottawa Scale
SBP: systolic blood pressure
TC: total cholesterol
WMD: weighted mean difference
